# Combining High-Pressure Processing and Supercritical Carbon Dioxide for Inactivation of *Listeria innocua*

**DOI:** 10.3390/foods12193563

**Published:** 2023-09-25

**Authors:** Bjørn Tore Rotabakk, Tone Mari Rode

**Affiliations:** Nofima AS—Norwegian Institute of Food, Fisheries and Aquaculture Research, PB 8034, NO-4068 Stavanger, Norway; tone.mari.rode@nofima.no

**Keywords:** high-pressure processing, supercritical carbon dioxide, *Listeria innocua*

## Abstract

The effect of high-pressure treatment with supercritical CO_2_ on the inactivation of *Listeria innocua* in a fish soup was investigated. The soup was inoculated with *L. innocua*, packaged in modified atmosphere with 50:50 or 95:5 CO_2_:N_2_, high-pressure processed (300, 350, 400 and 600 MPa, 2 min) under subcritical (T < 304 K) or supercritical conditions (T > 304 K) and stored at 4 °C for up to 53 days. Treatment at 400 and 600 MPa had a significant (*p* < 0.05) effect on *L. innocua* under both supercritical and subcritical conditions. In contrast, pressurization at 350 MPa and supercritical conditions were needed to significantly (*p* < 0.05) inactive *L. innocua*. Increased levels of CO_2_ in the headspace significantly (*p* < 0.05) reduced the bacterial load during processing, and supercritical conditions had a significant (*p* < 0.01) interaction with both CO_2_ levels and pressure. Increased storage time gave significantly increased levels of *L. innocua* at 400 and 600 MPa. In addition, high levels of CO_2_ significantly decreased (*p* < 0.001) growth. However, 350 MPa under supercritical conditions seemed to set the *L. innocua* in a permanent lag phase, with slow and steadily decreasing numbers of bacteria during storage. All the design variables resulted in significant inactivation of *L. innocua*, and supercritical conditions combined with high levels of CO_2_ inhibited the recovery of *L. innocua* to a large degree.

## 1. Introduction

*Listeria monocytogenes* is widespread and is a foodborne pathogen that can cause listeriosis. Listeriosis is a relatively rare but potentially fatal disease, especially for individuals with compromised immune systems, such as the elderly, pregnant women and newborns [1]. Even though *L monocytogenes* are easily killed by heat treatment, post-process cross-contamination from equipment and the environment is of great concern, and various strategies are used to control post-process contamination, including in-pack post-lethality treatments [2]. Several reports have concluded that high-pressure (HP) processing has a good inactivation effect on *L. monocytogenes* [3].

HP processing (HPP) is a preservation technology for food that can act as an alternative to thermal pasteurization and preserve the sensory and nutritional quality well [4]. HPP affects both microbiological and enzymatic activity, and a long shelf life can be obtained [5]. The effect of HPP is independent of the geometry and size of the food [6], and works almost instantly throughout the food [7]. Hence, HPP can be a faster processing method than traditional thermal processing. It has been shown that Gram-positive bacteria are more resistant to HPP [8] than Gram-negative bacteria. HPP does not affect primary bonds, such as covalent bonds. However, bonds responsible for maintaining the secondary, tertiary or quaternary structures of large molecules and complex organized structures are affected [9], which might explain why Gram-positive bacteria are more resistant to HPP than Gram-negative ones. It is common to use pressure levels from 400 to 600 MPa to obtain pasteurized food. However, using the highest pressure levels can lead to changes in color and texture, depending on the food and matrix. Combining HPP with other technologies can facilitate the use of a milder pressure treatment and positively affect the food quality. 

Carbon-dioxide-assisted high-pressure processing is a relatively new combination technique for reducing the microbial flora in real food systems [10]. CO_2_ has been used to inhibit or slow down bacterial growth for decades [11], and Gram-positive bacteria are more resistant to CO_2_ than Gram-negative ones [11,12]. One of the inhibiting mechanisms for CO_2_ is its ability to penetrate the bacteria’s cell wall and alter the intracellular pH [13]. CO_2_-assisted HPP has shown promising results on both Gram-negative and Gram-positive bacteria, such as *Staphylococcus aureus*, *Escherichia coli* [4], *Brochothrix thermosphacta*, *Salmonella* Enteritidis [6] and *L. innocua* [14]. It is also known that CO_2_-assisted HPP affects enzyme activity [15]. 

Another combination of CO_2_ and high pressure is supercritical CO_2_. Supercritical CO_2_ is characterized by liquid-like density and gas-like diffusion. The gas-like diffusion allows supercritical CO_2_ to diffuse through complex matrices quickly, and the liquid-like density confers a high extraction factor [16]. The bactericidal effect of supercritical CO_2_ is explained by acidification, disruption or fluidization of the cell membrane, inactivation of enzymes, and disordering of the electrolytic balance, among others [17]. Additionally, supercritical CO_2_ preserves the sensory and nutritional quality of the food, unlike thermal food processing [18]. Supercritical CO_2_ has been studied for decades, mainly at lower pressure levels and increased treatment time, typically 8 to 20 MPa for 30–120 min [19]. Long holding times have been pointed out as one of the challenges to making this process commercial and cost effective [18].

As it is pathogenic, *L. monocytogenes* can be a challenging organism to use in microbial studies. Several studies have been performed using the non-pathogenic *L. innocua* as a substitute, as *L. innocua* has high phenotypic similarity to *L monocytogenes* [20]. Studies have shown that the organisms have comparable behavior against high pressure [21], modified atmosphere, temperature and acidification [22,23]. It is also vital to perform inactivation studies in real food, as differences in pressure susceptibility have been detected for both *L. monocytogenes* and *L. innocua* in food versus buffered solutions [24].

The objective of this study was to investigate the inactivation of L. innocua in a fish soup packed in a modified atmosphere at two different CO_2_ levels (50 or 90%), processed at four different pressure levels (300, 350, 400 and 600 MPa) under supercritical or liquid CO_2_ conditions, and investigate if CO_2_-assisted HPP can enable the use of decreased pressures.

## 2. Materials and Methods

### 2.1. Design

A full factorial design with HPP (300, 350, 400 and 600 MPa), packaging conditions (50% and 95% CO_2_, balanced with N_2_) and state of CO_2_ (liquid or supercritical) as design variables was set up (Table 1). The responses in this trial were the reduction and growth of *L. innocua* in a milk-based fish soup, so before packaging, *L. innocua* was added to the soup. Supercritical conditions could, naturally, not be applied on the 0.1 MPa samples, as the pressure must be above 7.39 Kpa [16]. The design was carried out in two separate trials, with 0.1, 400 and 600 MPa in the first trial and 0.1, 300 and 350 MPa in the second. The experiments were performed by repeating the complete design twice, on two days for each test. Three technical replicates were used in all analyses unless otherwise stated.

### 2.2. Samples

The fish soup was diary based and made using a commercial product recipe. The ingredients and sterilization followed Rode et al. [14], but in short, the sterilization program was a $F_{121\;{}^{\circ}C}^{10\;{}^{\circ}C}\;$≥ 3 min process based on core temperature. The soup was stored at 1 °C before use. 

### 2.3. Bacterial Growth Conditions and Inoculation of the Fish Soup

*L. innocua* ATCC 33090, obtained from Oxoid (Hampshire, UK), was cultured and inoculated as described in detail by Rode et al. [14]. In short, the stock culture was maintained in Microbank (Pro-Lab Diagnostics, Richmond Hill, ON, Canada) at −80 °C. Before use, *L. innocua* was grown overnight before being re-cultured in several 200 mL flasks of TSA-YE, and incubated at 30 °C at 150 rpm for 20 h to a cell density of approximately 1 × 10^8^ cells mL^−1^. The bacteria were upconcentrated by centrifugation. A high initial concentration of *L. innocua* was chosen to detect possible differences between the packaging regimes. *L. innocua* was added to the fish soup, diluted 1:100, giving an initial concentration of approximately 1 × 10^8^ bacteria per ml soup.

### 2.4. Carbon Dioxide Packaging (CO_2_)

The 40 ± 2 g inoculated soup was packed in 120 mL HDPE trays (561, Barry Bebo Food Packaging, Kristiansand, Norway). The headspace was evacuated during packaging and subsequently flushed with a gas mix with high or low CO_2_ according to the design (Table 1) before heat sealing with a barrier top web film. Details about materials and transmission rates are described in [14], but a PA/PP top web and HDPE trays were used to pack the soup in a modified atmosphere. All packages were heavily agitated for 30 s after packaging to increase the mass transfer of CO_2_ into the product before HPP treatment.

### 2.5. Pressure Treatment

The HPP samples were pressurized for 2 min at 300, 350, 400 and 600 MPa in a high hydrostatic pressure machine QFP 2L-700 (Avure Technologies Inc., Columbus, OH, USA). The HPP samples were tempered in a water bath before treatment and processed at 10 or 22 °C to run the samples at one pressure level at both supercritical and non-supercritical conditions. The processing temperature was, on average, 23.5 ± 3.4 and 36.8 ± 2.8 °C when the pressure level was reached, securing supercritical conditions (above 304 K [31.1 °C] and 7.39 Kpa) for the latter at all pressures [25]. The come-up time was approximately 70, 77, 85 and 115 s for 300, 350, 400 and 600 MPa, respectively, whereas the pressure release was immediate. The duration of treatment did not include the come-up time. 

### 2.6. Storage and Sampling

Pressurized and non-pressurized samples were stored in a cooling cabinet at 4.0 ± 0.5 °C in the dark (Porkka CM 710 F, Huurre Group OY, Vantaa, Finland), and the temperature was logged for the entire storage period (Tracksense^®^Pro temperature loggers, Ellab AS, Hillerød, Denmark). All samples were analyzed before and after exposing the fish soup to HPP on days 0, 6, 13, 20 and 27 for samples processed at 300 and 350 MPa, and after 13, 27, 41 and 53 days of storage for samples processed at 400 and 600 MPa. Control samples were only analyzed on days 0, 6 and 13. During the storage study of up to 53 days, only samples not spoiled at the previous sampling point were analyzed.

### 2.7. Headspace Gas Composition

The headspace gas composition (CO_2_ and O_2_, %) was assessed using an oxygen and carbon dioxide analyzer (Checkmate 9900 analyzer, PBI-Dansensor, Ringsted, Denmark). After the top web intrusion, an aliquot (20 mL) of the headspace in empty boxes was collected with a syringe. A foam rubber septum (Nordic Supply, Skodje, Norway) was used to avoid introducing a false atmosphere into the gas analyzer.

### 2.8. pH Analysis

The pH was measured directly in the fish soup samples (Mettler Toledo Go Pro pH meter, Mettler Toledo, Columbus, OH, USA).

### 2.9. Microbial Analysis

Microbial analyses were performed according to Rode et al. [14], where, in short, *L. innocua* was quantified (colony forming units (CFU) g^−1^) by surface plating on tryptic soy agar (Oxoid) with 0.6% yeast extract (Merck) (TSA-YE), and incubation at 30 °C for 48 h. A mechanical spiral plater (Eddy Jet, IUL Instruments, Barcelona, Spain) was primarily used. Some manual plating was performed for the low dilutions. 

### 2.10. Statistical Analyses

The setup was a full factorial design, and general linear modeling (GLM) and one-way ANOVA were used to determine statistically significant main and interaction effects of the different parameters (Minitab^®^ Statistical Software v17, Minitab Ltd., Coventry, UK) using Tukey’s HSD test at level *p* < 0.05 (95%). ANOVA was applied to log_10_ transformations of the microbiological counts. The results are presented as Mean ± Std Dev, if not stated otherwise.

## 3. Results and Discussion

The design was implemented in two separate trials, with 0.1, 400 and 600 MPa in the first trial and 0.1, 300 and 350 MPa in the second. Significant differences (*p* < 0.02, ANOVA) in CO_2_ levels were found when comparing the initial gas mix in the two trials, ending up with 94.6 ± 0.4 and 48.5 ± 0.4% CO_2_ in the first (400 and 600 MPa) and 96.2 ± 1.1 and 47.9 ± 0.7% CO_2_ in the second trial (300 and 350 MPa). However, differences of 1.6 and 0.6% were considered ignorable when applying such high levels of CO_2_ and deemed not to affect the results. No significant (*p* > 0.08, ANOVA) differences in O_2_ levels were detected, ending with 0.41 ± 0.14% O_2_ in the initial gas mix for all variants. The initial starting concentration of *L. innocua* in the soup, before any treatment, was, on average, 8.64 ± 0.19 log_10_ CFU/g. No significant (*p* = 0.056, ANOVA) difference in pH between the two trials was found, giving an average starting pH of 5.70 ± 0.15. The results of both trials and repetitions were then combined into one data set, and will be discussed as one trial, giving n = 6 for most of the samples.

### 3.1. Reduction of L. innocua in Fish Soup after HPP

**Figure 1:**
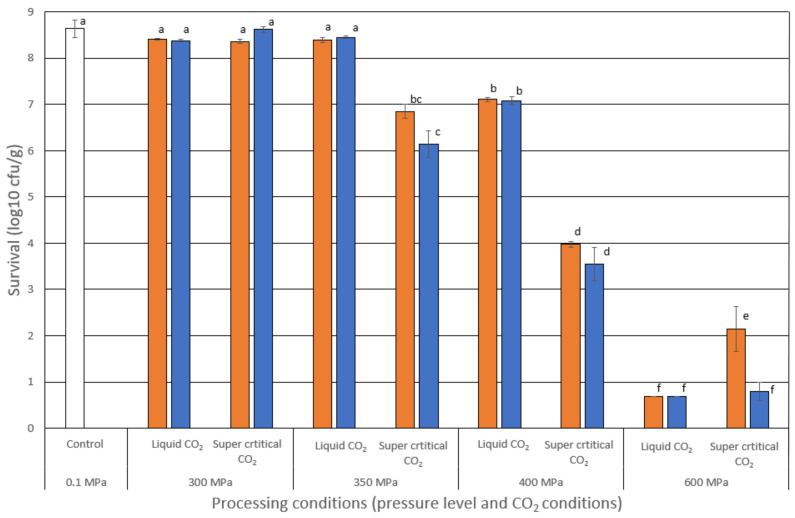
Survival of *L. innocua* (log_10_ CFU/g) in the fish soup right after high-pressure processing (0.1, 300, 350, 400 or 600 MPa) at day 0. The soup was packaged in a modified atmosphere with 50% CO_2_ (orange bars) or 95% CO_2_ (blue bars) in the packaging gas before processing. The temperature was set to give supercritical CO_2_ in half the number of runs. The white bar represents the initial start level of bacteria in the soup (Control). Bacterial counts were measured on TSA-YE plates (n = 6). The standard errors of means are shown as whiskers. The detection limit was 10 CFU/g. Columns with different lower superscripts are significantly (*p* < 0.05) compared via one-way ANOVA and Tukey’s HSD test.

A clear pattern evolved when examining the reduction of *L. innocua* measured immediately after processing. All the design variables significantly impacted the reduction of *L. innocua* when analyzing the main factors. The factor explaining most of the variance was as expected to be the pressure level (*p* < 0.001, F = 990, GLM), where all HPP treatments, except 300 MPa, produced a significantly (*p* < 0.05, Tukey) stepwise increasing reduction in *L. innocua* with increasing pressure (Figure 1). In a previous study on different packaging regimes, we reported the reduction of *L. innocua* in fish soup to be 3.5 and 7.3 log_10_ CFU/g at 400 and 600 MPa [14]. Others, like Yuste et al. [26], have found a 3.46 log_10_ CFU/g reduction of *L. innocua* at 400 MPa in poultry meat. Additionally, an *L. innocua* cocktail showed no surviving cells in minced trout added 1% NaCl at 500 MPa in 2 min, with a starting level of above 8 log_10_ CFU/g [27]. Samples without salt gave a reduction of 5.6 log_10_ CFU/g. The amount of salt in the present study was 0.66%. All of these results are comparable to the reduction observed in the present study.

Both supercritical conditions (*p* < 0.001, F = 106, GLM) and high CO_2_ levels (*p* = 0.010, F = 7.05, GLM) significantly reduced the bacterial load during processing. Further knowledge was obtained by studying the interactions of the factors. Supercritical conditions had significant interactions with both pressure (*p* < 0.001, F = 87, GLM) and CO_2_ level (*p* = 0.009, F = 7.2, GLM). Supercritical CO_2_ significantly reduced the bacterial load when processing at 350 and 400 MPa (Figure 1), while no such effect was observed at 300 and 600 MPa. Additionally, high levels of CO_2_ only had an impact under supercritical conditions. No other significant interactions were detected (*p* > 0.071, GLM). This implies that both supercritical conditions and high levels of CO_2_ significantly increase the reduction of *L. innocua* at 350 and 400 MPa. In comparison, 600 MPa gave such large effects that no additional impact of elevated CO_2_ and supercritical CO_2_ was detectable.

CO_2_-assisted HPP has been reported to give mixed results. Al-Nehlawi et al. (2012) used pure CO_2_ in the headspace in MAP combined with HPP at 350 MPa for 10 min, obtaining about a 2.5 log_10_ CFU/g reduction of *L. innocua* in poultry sausages, while packaging with air only gave a decrease of 0.5 log_10_ CFU/g [6]. In another study, we reported that 400 MPa for 2 min at 20 °C combined with CO_2_ showed no additional reduction of *L. innocua* compared to vacuum in fish soup [14]. Additionally, 150 MPa for 30 min at 1 °C combined with 50% CO_2_–50% N_2_ gave a log_10_ 2.5 reduction in *L. monocytogenes* inoculated on salmon [10]. None of these studies studied the effect of supercritical CO_2_. Reductions in bacterial levels and sterilization as an effect of supercritical CO_2_ have mainly been studied at lower pressure levels and increased treatment times, typically 8 to 20 MPa and 30 to 120 min [19]. Supercritical CO_2_ treatment of shrimp gave a log_10_ 2 (99%) reduction in *L. monocytogenes* at 13.7 MPa and 35 °C for 2 h [28], while a log_10_ 7 reduction was observed on alfalfa sprout seeds at 20 MPa and 45 °C for 15 min [29]. It has been shown that the efficiency of supercritical CO_2_ treatment is related to the diffusion time for CO_2_ into the microbial cells, which is directly related to temperature, food matrix, time and pressure [30]. CO_2_ was already dissolved into the soup by agitating the trays, exposing the inoculated *L. innocua* to the supercritical CO_2_ instantly. However, dissolving into the microbes is still related to time, and it is believed to be likely that the effect of both CO_2_-assisted HPP and supercritical CO_2_ could be observed at 300 MPa in the present study if the holding time were increased. However, by increasing the pressure, adequate inactivation of *L. innocua* is achievable within minutes.

### 3.2. Regrowth of L. innocua during Storage

**Figure 2:**
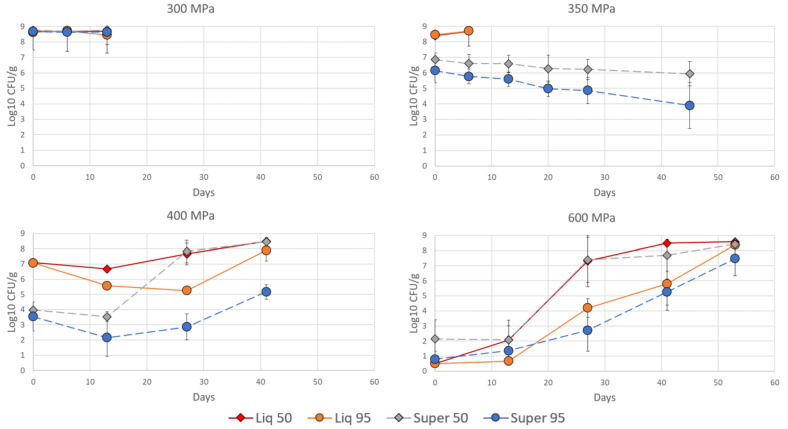
Growth of *L. innocua* in fish soup. The fish soup was exposed to different pressure levels (300, 350, 400 and 600 MPa), liquid CO_2_ (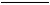) or supercritical CO_2_ (- - - -) and stored in a modified atmosphere with 50% CO_2_ (♦) or 95% CO_2_ (○) in the initial gas mix for up to 53 days. The soup was stored at 4.0 ± 0.5 °C. Bacterial counts (log_10_ CFU/g) were measured in TSA-YE plates, and means ± std dev are shown.

*Listeria* grows well under refrigerated temperatures and can maintain growth after exposure to both CO_2_ and HPP. The regrowth of *L. innocua* during the storage period is shown in Figure 2. Again, pressure had the highest explanation of the observed variance, with a significant decrease in *L. innocua* with increasing pressure (*p* < 0.001, F = 339, GLM). As expected, increased storage time significantly increased the total level of *L. innocua* (*p* < 0.001, F = 109, GLM) in the trials. Both non-treated samples (0.1 MPa) and samples treated at 300 MPa were overgrown at day 13 (data not shown), while only the 600 MPa samples were analyzed at day 53. In addition, a significant decreasing effect of supercritical conditions (*p* < 0.001, F = 80.5, GLM) was detected, while increased CO_2_ in the packaging gas significantly slowed the growth (*p* < 0.001, F = 65.2, GLM).

Both supercritical conditions (*p* < 0.001, F = 32.4, GLM) and the CO_2_ level (*p* < 0.001, F = 15.3, GLM) had a significant interaction with HPP. This can be clearly observed when studying the growth curves after each HPP pressure (Figure 2); there was no significant difference between liquid CO_2_ (continuous lines) and supercritical CO_2_ (dashed lines) (*p* = 0.833, ANOVA) in samples treated at 600 MPa (independent of CO_2_ level). However, supercritical CO_2_ significantly (*p* < 0.001, ANOVA) reduced the growth of *L. innocua* in samples treated at 350 and 400 MPa. Additionally, high levels of CO_2_ significantly (*p* < 0.001, ANOVA) slowed the growth of *L. innocua* compared to low levels of CO_2_ with increasing pressure. None of the significant effects described above could be observed on any samples subjected to 300 MPa treatment. 

Increased levels of CO_2_ resulted in a small but significant (*p* < 0.001, F = 17.3, GLM) decrease in pH, from 5.80 ± 0.25 in 50% CO_2_ to 5.72 ± 0.22 in 95% CO_2_. Additionally, increasing pressure resulted in significantly (*p* < 0.001, F = 21.3, GLM) increased pH (Table 2). Including storage days as a covariate gave no vital information (*p* = 0.876, GLM). No other significant effects were found.

*Listeria* is usually little affected by CO_2_ in modified atmosphere packages [12,31], but this study shows that the high levels of CO_2_ during storage slow down its growth. Farber et al. [13] found a lag phase of 11 to 13 days of *L. monocytogenes* packaged with 90% CO_2_ and stored at 4 °C, which is in agreement with the results for all of the 600 MPa variants in this study. However, the lag phase increases with decreasing pressure for the variants with high CO_2_. More precisely, 350 MPa under supercritical conditions seems to set the *L. innouca* in a permanent lag phase, with slowly but steadily decreasing numbers of bacteria. This phenomenon might imply that even if supercritical conditions at 350 MPa for 2 min do not kill all the *L. innouca*, they get injured and do not manage to start growing, even after 45 days. Processing at 400 and 600 MPa gave a high initial bacterial reduction and an additional reduction/no change in bacterial numbers of *L. innocua* up to day 13. Two weeks later, the surviving cells multiplied when supercritical CO_2_ was applied. One hypothesis might be that the *Listeria* bacteria that survive pressure levels at 400 MPa or above immediately enter a mode of action where some specific repairing mechanisms are initiated. For the *Listeria* bacteria exposed to lower pressure levels, i.e., 350 MPa or below, in combination with supercritical CO_2_, it appeared that the bacteria entered a stage in which they could not grow and were waiting for conditions to improve. Damage caused by the pressure load (below 350 MPa), in addition to persistent CO_2_ and low pH, did not seem to initiate the same repair mechanisms as bacteria exposed to pressures above 400 MPa. Over time, the bacteria gradually started to die. This was in contrast to the bacteria exposed to pressures ≥ 400 MPa, which exhibited a high initial reduction but started to multiply, and showed increased bacterial numbers at day 13.

It has been shown that decreasing pH from 6.0 to 5.5 increased the lag phase of *L. innocua* by 3.4 h [32], but the observed pH effect of both high CO_2_ levels and different pressures alone (Table 2) cannot explain the observed difference. One possible explanation for this phenomenon could be that several enzymes are affected by the high pressure and supercritical CO_2_. It is known that high-pressure-induced enzyme inactivation is a very complex phenomenon and that the nature of enzymes is unpredictable due to exposure to very high pressure (reviewed by Chakraborty et al. [33]). Supercritical CO_2_ has been shown to alter enzymatic activity functions and alter the proteins’ three-dimensional structure [19]. Additionally, internal pH affects enzyme activity. A summary of these hurdles indicates that exposure to 350 MPa under supercritical conditions may have affected either some enzyme in *L. innocua* or enzymes in the fish soup, resulting in an inhibitory effect on *L. innocua*, such that growth was inhibited. Further research is needed to identify what causes this effect. It includes other microbiota to see if this effect is species dependent or if this combination of pressure and supercritical CO_2_ generally inhibits several microorganisms.

## 4. Conclusions

CO_2_-assisted HPP has a positive effect in reducing the amount of *L. innocua* in fish soup. Processing under supercritical CO_2_ conditions enhanced this effect. Additionally, high levels of CO_2_ in the headspace increased the bacterial reduction during HPP and decreased the bacterial growth during storage. *Listeria* can grow at refrigerated temperatures and recover after high-pressure treatment, so finding process and storage protocols that ensure food safety is essential. Combining CO_2_ and HPP, especially under supercritical conditions, resulted in the recovery of *L. innocua* being inhibited to a large degree, and showed that decreased pressures can be used. 

## Figures and Tables

**Table 1:** Full factorial design ^1^, where “pressure”, “state of CO_2_”, and “level of CO_2_ in packaging gas” are the factors in the experimental setup.

Design Variable	Levels				
Pressure (MPa)	0.1	300	350	400	600
State of CO_2_	Liquid	Supercritical ^1^			
Level of CO_2_ in packaging gas (%)	50	95			

^1^ Supercritical conditions could only be applied on samples pressurized above 7.4 Kpa.

**Table 2:** Effect of high-pressure processing on pH in a fish soup inoculated with *Listeria innocua*. Superscript letters indicate statistically significant (*p* < 0.05) differences.

Pressure (MPa)	pH
0.1	5.32 ± 0.19 ^a^
300	5.42 ± 0.14 ^a^
350	5.74 ± 0.15 ^b^
400	5.89 ± 0.24 ^c^
600	5.92 ± 0.23 ^c^

## Data Availability

The data presented in this study are available on request from the corresponding author.
